# Decoding Prognostic Signatures in Brain Metastatic Non-Small-Cell Lung Cancer via Integrated Multi-Omics and Network Analysis

**DOI:** 10.3390/ijms27083598

**Published:** 2026-04-17

**Authors:** Prithvi Singh, Ravins Dohare, Tarique Sarwar, Hajed Obaid A. Alharbi, Arshad Husain Rahmani

**Affiliations:** 1 Centre for Interdisciplinary Research in Basic Sciences, Jamia Millia Islamia, New Delhi 110025, India; prithvi.mastermind@gmail.com; 2 Department of Medical Laboratories, College of Applied Medical Sciences, Qassim University, Buraydah 51452, Saudi Arabia; t.sarwar@qu.edu.sa (T.S.); hajed.alharbi@qu.edu.sa (H.O.A.A.)

**Keywords:** Non-small cell lung cancer, brain metastasis, prognosis, pathogenesis

## Abstract

Non-small-cell lung cancer (NSCLC) constitutes approximately all lung cancers (LCs), and metastasis remains a major challenge in its treatment, thus necessitating the detection of novel molecular players involved in this process. In this study, we performed a comprehensive analysis of microarray and RNA-seq cohorts extracted from The Cancer Genome Atlas (TCGA) and Gene Expression Omnibus (GEO) to identify differentially expressed genes (DEGs) and differentially expressed miRNAs (DEMs) and associated them with metastasis-related genes involved in brain metastasis (BM) in NSCLC. We thus identified differentially expressed metastatic genes (DEMGs) and constructed a protein–protein interaction network (PPIN) using these DEMGs. These DEMGs were further analyzed for associations with patient age, gender, and tumor stage, and the significant impact of specific genes on overall survival (OS) was assessed to determine the prognostic significance of the identified targets. We finally constructed a three-node microRNA (miRNA) feed-forward loop (FFL) involving miR-23b-3p, CD44, and five transcription factors (TFs) [EOMES, FOS, FOSL1, GLIS3, TP63] specific to NSCLC metastasis. Further mutational analysis of these FFL elements revealed that all were altered in the patient samples analyzed. Thus, our study identified potential genomic drivers that may play crucial roles in NSCLC BM. Overall, it provides valuable insights for the discovery of novel therapeutic targets in the management of NSCLC metastasis. However, further in vitro and in vivo experimentations are needed to justify the prognostic role of NSCLC biomarkers in BM pathogenesis.

## 1. Introduction

Lung cancer (LC) ranks among the most frequently diagnosed cancers and is a leading cause of cancer-associated morbidity and mortality. It is further classified into small-cell lung cancer (SCLC) and non-small-cell lung cancer (NSCLC). NSCLC is the most common subtype, accounting for approximately 80% of newly diagnosed LC cases [1]. It further encompasses a heterogeneous class of histomorphological tumor subtypes [2]. The predominant subtypes of NSCLC are adenocarcinoma, accounting for most cases, and squamous cell carcinoma, accounting for a smaller proportion [1,3]. The major risk factors for developing NSCLC are tobacco smoking, radon exposure and air pollution [4,5].

Clinically, only a few NSCLC patients get diagnosed at early stages (stage I or II), facilitating treatment by surgical resection. However, most patients are confronted with an advanced stage (stage IV), when the tumor has already metastasized, and surgical resection is difficult. The major challenge is with patients presenting with brain metastases (BM), with most patients being diagnosed with BM within two years after diagnosis of the primary tumor [6,7]. Conversely, NSCLC accounts for almost half of all the cases diagnosed with BM and is associated with a poor prognosis, having a median survival of only months if left untreated [8,9]. Many factors play an important role in determining the prognosis of such patients, including age, the number of BM and primary tumor subtype [10]. Surgical resection may be high risk in cases with BM, while whole-brain and stereotactic radiotherapies, along with local control for BM, may prove effective, although the potential for radionecrosis and neurotoxicity is a major concern in one-third of patients [11,12,13]. Furthermore, treatment using chemotherapeutic drugs is not effective due to their inability to cross the blood–brain barrier (BBB). The reported response rates to chemotherapy in BM range from months to years, with overall survival (OS) also ranging from months to years [14,15]. Thus, exploring novel strategies to combat NSCLC cases with BM is pivotal.

In recent years, we have advanced our understanding of the molecular mechanisms underlying BM in NSCLC patients. Dedicated efforts by clinicians and researchers have identified gene mutations responsible for NSCLC, including those in *EGFR*, *ROS1*, *ALK*, and *BRAF*. Following the identification of these mutations, several lines of tyrosine kinase inhibitors (TKIs) have been developed as therapeutic strategies for NSCLC [16,17,18]. In NSCLC patients with BM harboring *EGFR* mutations, TKIs such as gefitinib, erlotinib and afatinab have been used with response rates and OS of several months, thus demonstrating improved therapeutic outcomes [6,19,20]. In addition, immune checkpoint inhibitors (ICIs) have also emerged as a game-changer in the NSCLC treatment, significantly improving patients’ OS [21]. However, their safety and efficacy in treating BM cases are still under evaluation, especially given concerns about their ability to cross the BBB.

Nevertheless, all these efforts have significantly improved the OS of NSCLC patients. However, despite this progress, the median survival rate for NSCLC patients with BM remains quite low, underscoring the importance of improved patient stratification. To this end, identifying novel molecular biomarkers associated with BM in NSCLC can help devise novel treatment strategies. The present study addresses the gaps in our understanding of the molecular basis of BM in NSCLC patients. While recent literature has identified general “key genes” associated with BM, these studies often lack the regulatory context necessary to understand how these genes are controlled or how they interact within a functional network. To this end, we performed a comprehensive analysis encompassing lung adenocarcinoma (LUAD) and lung squamous cell carcinoma (LUSC) RNA-seq cohorts from The Cancer Genome Atlas (TCGA), followed by the identification of differentially expressed genes (DEGs)/differentially expressed microRNAs (DEMs). We uniquely overlapped these findings with BM-DEGs obtained from a dedicated Gene Expression Omnibus (GEO) dataset to identify differentially expressed metastatic genes (DEMGs). By requiring consistency across both independent cohorts and distinct technological platforms (RNA-seq and microarray), our study implements a rigorous biological filter that minimizes the false positive noise typical of single-dataset bioinformatics reports. Moving beyond simple gene-list identification, we subsequently determined the role of molecular signatures and their prognostic implications on clinical outcome. The major novelty and distinct contribution of our work lie in the construction of an NSCLC metastasis-specific three-node miRNA feed-forward loop (FFL) comprising *CD44*, miR-23b-3p, and five transcription factors (TFs) [*EOMES*, *FOS*, *FOSL1*, *GLIS3*, *TP63*]. This is a first-of-its-kind study that bridges the gap between primary NSCLC and BM patients through the lens of integrated regulatory networks, offering a more granular roadmap for targeted therapeutic intervention. However, further in vitro and in vivo experiments are needed to establish the role of NSCLC biomarkers in BM pathogenesis.

## 2. Results

### 2.1. Data Extraction and Differential Expression Analysis (DEA) of Microarray and RNA-Seq Cohorts

As per the inclusion and exclusion criteria outlined in the methodology, we extracted LUAD and LUSC cohorts from UCSC Xena comprising a total of 386 and 347 stage-wise tumor patient samples. Table 1 and Table 2 summarize the clinical information associated with LUAD and LUSC cohorts. Again, as per the inclusion and exclusion criteria specified, we extracted messenger RNA (mRNA) expression profile from GEO possessing accession number GSE161116 with 28 patient samples. The patient samples were further distributed across different stages for both LUAD and LUSC cohorts. For LUAD, stages I to IV comprised 217, 89, 62, and 18 patient samples, respectively, while for LUSC, stages I to IV comprised 173, 121, 50, and 3 patient samples, respectively. The total number of upregulated and downregulated DEGs/DEMs in both LUAD and LUSC cohorts is summarized in Table 3 and Table 4. In microarray analysis, we identified 200 DEGs (95 downregulated + 105 upregulated) as per the above-mentioned threshold.

### 2.2. Collection of DEMGs

**Figure 1:**
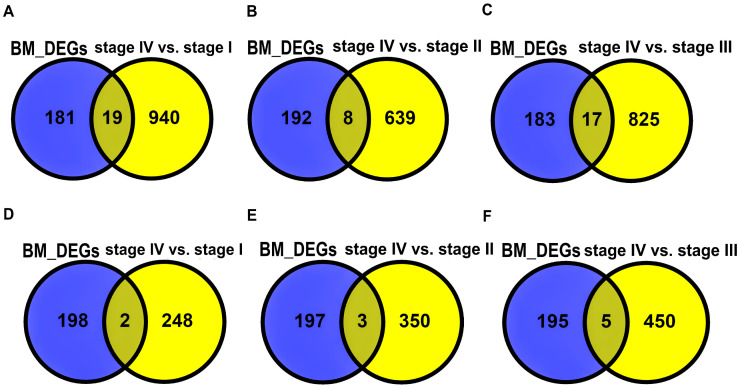
Venn plot showing overlapping DEMGs across the LUAD cohort (**A**–**C**) and the LUSC cohort (**D**–**F**). The yellow- and blue-colored areas represents gene sets pertaining to stage-wise (i.e., stage IV vs. stage I, stage IV vs, stage II, stage IV vs. stage III) LUAD/LUSC DEGs and BM DEGs, respectively.

We performed an overlap analysis between the BM DEGs from the GEO dataset and the DEGs identified in the LUAD and LUSC cohorts to obtain DEMGs. These DEMGs represent genes that are differentially expressed in LC and BM. The Venn plots in Figure 1 show DEMGs specific to LUAD and LUSC cohorts. The age and gender information for stage-wise DEGs across the LUAD and LUSC cohorts is shown in Table 5. 

### 2.3. Protein–Protein Interaction Network (PPIN) and OS Analyses

**Figure 2:**
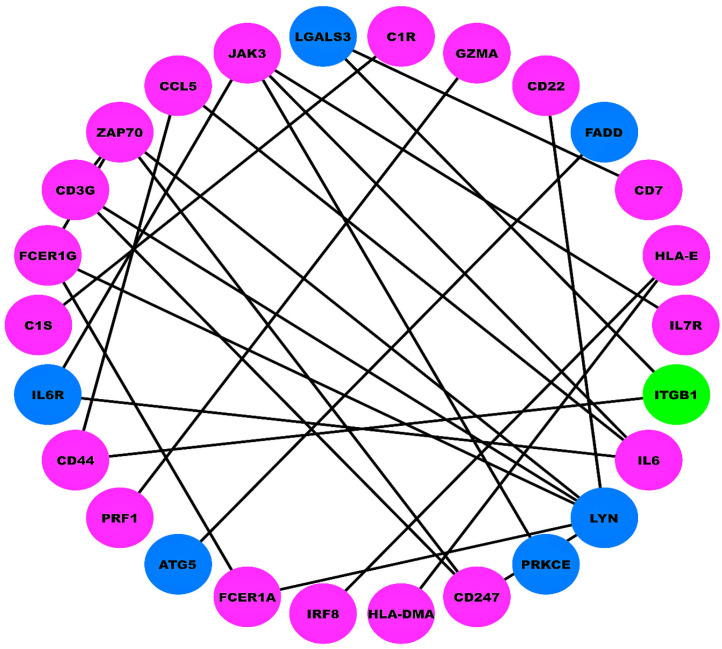
Unweighted and undirected PPI network comprising 26 nodes and 26 edges corresponding to STRING interaction score >0.9. Magenta-colored nodes represent downregulation status of DEGs, blue-colored nodes represent upregulation status of DEGs, and the green-colored node represents mixed status of DEGs (i.e., upregulation in one cohort and downregulation in another cohort).

**Figure 3:**
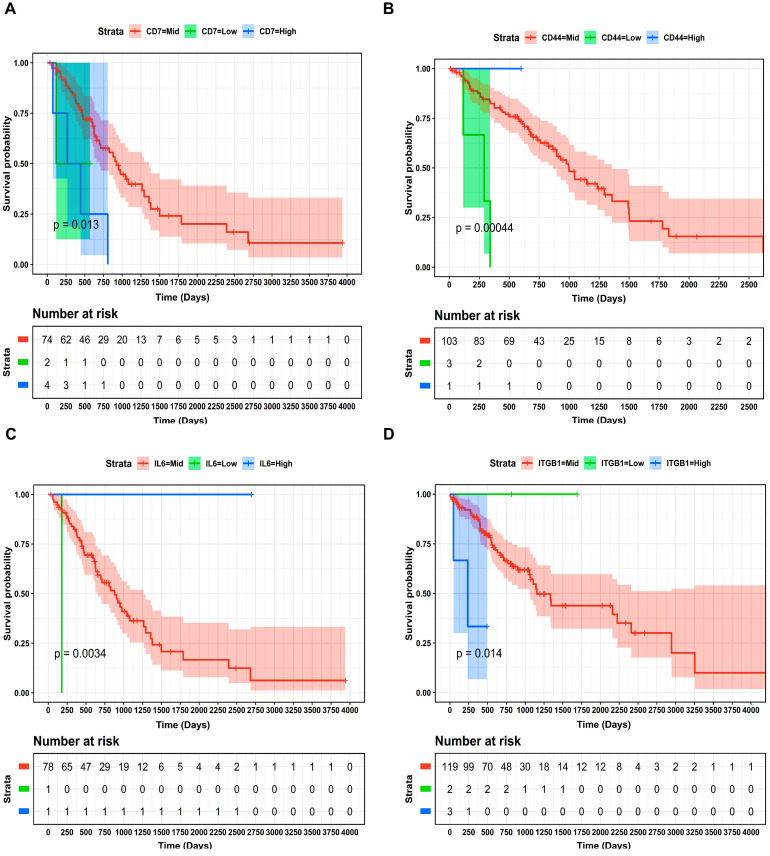
KM plots showing the OS of (**A**) CD7, (**B**) CD44, and (**C**) IL6 across the TCGA LUAD cohort. A KM plot showing OS of (**D**) ITGB1 across the TCGA LUSC cohort. The low-, mid-, and high-expression groups based on z-score were signified by green-, red-, and blue-colored curves. The number-at-risk table is presented at the bottom of each KM plot, and time is represented in days.

**Figure 4:**
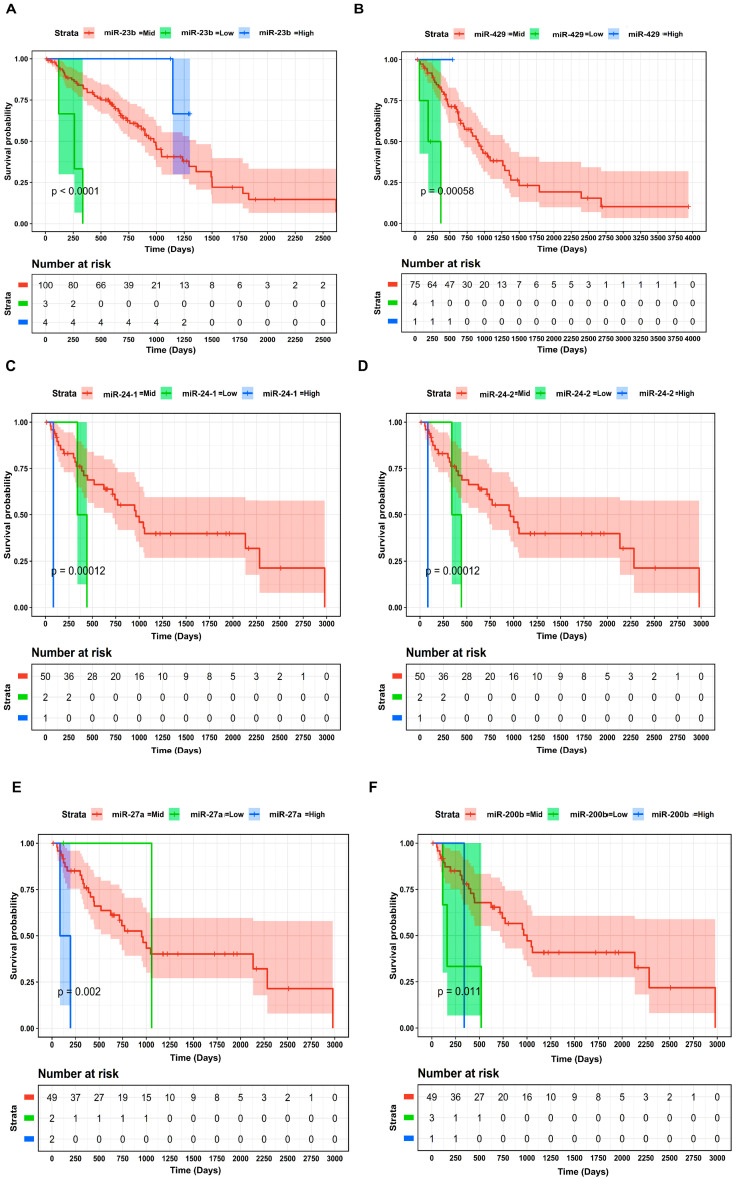
KM plots showing the OS of (**A**) miR-23b and (**B**) miR-429 across the TCGA LUAD cohort. KM plot showing OS of (**C**) miR-24-1, (**D**) miR-24-2, (**E**) miR-27a, and (**F**) miR-200b across the TCGA LUSC cohort. The low-, mid-, and high-expression groups based on z-score were signified by green-, red-, and blue-colored curves. The number-at-risk table is presented at the bottom of each KM plot, and time is represented in days.

A union comprising 49 DEMGs within the LUAD and LUSC cohorts was submitted to STRING for PPIN construction. Corresponding to an interaction score >0.9, the PPIN comprised 26 nodes and 26 edges, as shown in Figure 2. The Kaplan–Meier (KM) plots depicting the significant OS trends for DEMGs involved in PPIN and for all DEMs across the LUAD and LUSC cohorts are shown in Figure 3 and Figure 4, respectively. We found that lower expression levels of *CD44* (stage IV vs. stage II), *IL6* (stage IV vs. stage III), *CD7* (stage IV vs. stage III), miR-23b (stage IV vs. stage II) and miR-429 (stage IV vs. stage III) worsened the OS in LUAD patients. In addition, higher expression levels of *ITGB1* (stage IV vs. stage II), miR-27a (stage IV vs. stage III), miR-24-1 (stage IV vs. stage III), miR-24-2 (stage IV vs. stage III), and lower expression levels of miR-200b (stage IV vs. stage III) worsened the OS of LUSC patients.

### 2.4. NSCLC Metastatic-Specific Three-Node miRNA FFL Analysis

#### 2.4.1. TF-mRNA Regulation

We compiled a total of five human TFs associated with *CD44* (only prognostically significant DEMG of interest identified from the above analysis). These TFs were *EOMES*, *FOS*, *FOSL1*, *GLIS3*, *TP63* and were selected based on a significant threshold corresponding to a p-value<0.01. These TFs were further validated through existing literature.

#### 2.4.2. miRNA-mRNA/TF Repression

**Figure 5:**
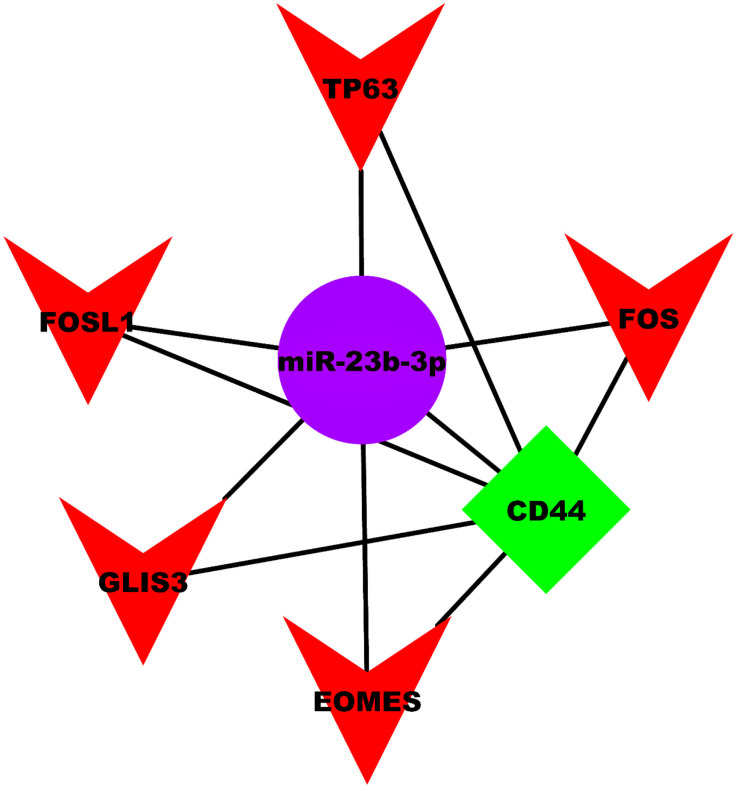
NSCLC metastatic-specific three-node FFL comprising seven nodes and eleven edges. It comprises one miRNA (miR-23b-3p), one gene (CD44), and five TFs (EOMES, FOS, FOSL1, GLIS3, TP63). Red triangular nodes signify NSCLC-specific TFs, green-colored diamond nodes signify prognostically significant DEMG, and purple-colored circular nodes signify prognostically significant DEM. Both CD44 and miR-23b-3p were present in stage IV vs. stage II groups of the LUAD cohort.

We further identified that, among all prognostic DEMs associated with LUAD and LUSC cohorts, only miR-23b-3p interacted with *CD44* and the five aforementioned TFs. The three-node miRNA FFL representing NSCLC metastasis was finally constructed based on interactions among *CD44*, the five identified TFs, and miR-23b-3p. Figure 5 shows the visual representation of the constructed FFL network. As depicted, the constructed NSCLC metastatic-specific three-node miRNA FFL comprised seven nodes and eleven edges.

### 2.5. Mutational Analysis of FFL Elements

**Figure 6:**
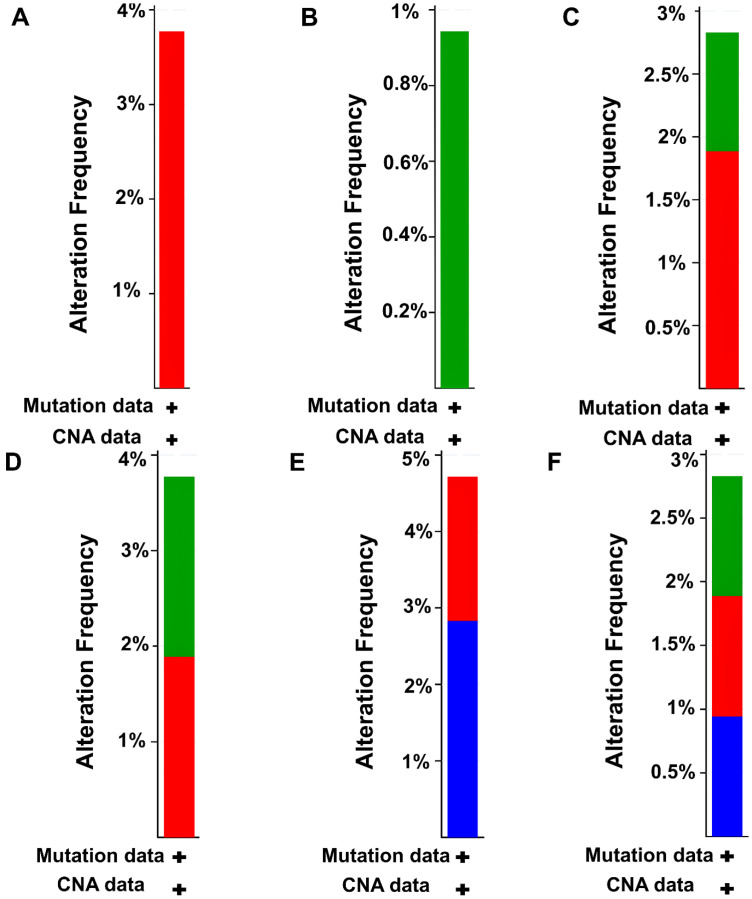
Bar plots showing alteration frequencies of (**A**) CD44, (**B**) EOMES, (**C**) FOS, (**D**) FOSL1, (**E**) GLIS3, and (**F**) TP63 across 107 TCGA LUAD patient samples (stage IV vs. stage II only). Red-, blue-, and green-colored shaded areas within each bar indicate amplifications, deep deletions, and missense mutations, respectively.

We further focused on the mutational analysis of the elements of our constructed FFL, including *CD44* and the five TFs. Given that *CD44* was more frequently expressed in stage IV vs. stage II samples in the LUAD cohort, we selected 107 patient samples from the LUAD (TCGA, Firehose Legacy) cohort in cBioPortal for further analysis. We thus submitted *CD44*, *EOMES*, *FOS*, *FOSL1*, *GLIS3*, and *TP63* as inputs. All six queried items were altered in 15% (16/107) of the analyzed patient samples. Specifically, *CD44*, *EOMES*, *FOS*, *FOSL1*, *GLIS3*, and *TP63* reported mutation frequencies of 4%, 0.9%, 2.8%, 4%, 5%, and 2.8% respectively. Bar plots shown in Figure 6 represent overall alteration frequencies of these elements based on the cancer-type summary analysis. *CD44*, *FOS*, *FOSL1*, *GLIS3*, and *TP63* showed 3.7% (4/107 cases), 1.8% (2/107 cases), 1.8% (2/107 cases), 1.8% (2/107 cases), and 0.9% (1/107 case) amplification frequencies, respectively. In addition, *EOMES*, *FOS*, *FOSL1*, and *TP63* showed 0.9% (1/107 case), 0.9% (1/107 case), 1.8% (2/107 cases), and  0.9% (1/107 case) missense mutation frequencies. *GLIS3* and *TP63* showed 2.8% (3/107 cases) and 0.9% (1/107 case) deep deletion frequencies.

## 3. Discussion

BM is a major clinical challenge and a leading cause of mortality in cancer patients. Even with a small size of metastatic lesion, BM is often associated with significant neurological damage, leading to poor prognosis marked with short median OS. Out of the various primary sources of tumor that can give rise to BM, lung, breast, and melanoma are the three most common causes, accounting for BM incidences [22]. Among these, LC is a significant contributor to BM as about 40–50% of patients diagnosed with LC are associated with BM [23]. Furthermore, NSCLC accounts for almost 50% of the total BM cases. Therefore, performing a separate analysis in NSCLC patients with BM is imperative. In addition, several prognostic factors such as age, the time between tumor diagnosis and whole-brain radiotherapy, and extracranial metastases have been associated with NSCLC patients with BM [24]. However, once cancer has spread to the brain, the treatment strategies become severely limited. Our limited understanding of the molecular mechanisms underlying BM has further hindered our ability to address this problem. Thus, given the high prevalence of BM in NSCLC patients, with high mortality and morbidity, there is a pressing need to identify genomic and transcriptomic biomarkers involved in NSCLC-BM and to correlate them with clinicopathological factors and patient outcomes. This will help us to identify prognostic biomarkers for patient stratification and therapeutic intervention.

In this study, we conducted a comprehensive analysis to elucidate the molecular basis of BM in NSCLC. We used the UCSC Xena browser to extract RNA-seq data from the TCGA-LUAD and LUSC cohorts and queried the National Center for Biotechnology Information (NCBI)-GEO for microarray data on NSCLC and BM. We conducted DEA to identify DEGs and DEMs (Table 3 and Table 4) between different cancer stage categories (i.e., stage IV vs. stage III, stage IV vs. stage II, stage IV vs. stage I) from both LUAD and LUSC cohorts. Later, we also conducted DEA to identify 200 DEGs between NSCLC and BM patients from GEO. The DEGs from each stage-wise category were overlapped with GEO DEGs to identify DEMGs associated with BM in NSCLC patients. The union of 49 DEMGs were identified as candidate genes associated with BM and subjected to PPIN analysis. Finally, through comprehensive OS analysis, the potential biomarkers associated with patient survival outcomes were revealed. To this end, the association of *CD44*, *IL6*, *CD7*, *ITGB1*, miR-23b, miR-429, miR-27a, miR-24-1, and miR-24-2 with OS was analyzed. Lower expression levels of *CD44*, *IL6*, *CD7*, miR-23b, and miR-429 were associated with worse OS in LUAD patients. Conversely, higher expression levels of *ITGB1*, miR-27a, miR-24-1, and miR-24-2 were associated with poor prognosis in LUSC patients. Furthermore, through the construction of an NSCLC BM-specific three-node miRNA FFL, we highlighted the intertwined molecular relationships among *CD44*, miR-23b-3p, and the five TFs (i.e., *EOMES*, *FOS*, *FOSL1*, *GLIS3*, *TP63*). The mutational analysis of these FFL elements unravels the molecular underpinnings of NSCLC BM. This information is useful to clinicians developing targeted therapies for NSCLC.

*CD44* is a marker of cancer stem cells and also plays an important role in cellular signaling. Its increased expression is usually associated with increased invasion, metastasis and cancer cell proliferation [25]. However, the relationship between *CD44* expression and OS varies across cancers. In the present study, we found lower *CD44* expression in stage IV vs. stage II, which was further associated with poor patient outcome in LUAD patients. Notably, previous studies show that cancer tissues express various *CD44* isoforms which differ according to tumor type and metastasis status. For instance, CD44s, an isoform of CD44, is downregulated in metastatic human tissue compared to non-metastatic tissue [26]. We also identified *CD7* and associated its expression with patient OS. *CD7* is a transmembrane glycoprotein known to be expressed on T cells, thymocytes, natural killer (NK) cells and immature subpopulations of B and myeloid cells [27]. It has been used as a lymphoid marker and linked with poor prognosis in myeloid malignancies [28]. In our case, we found lower *CD7* expression in stage IV vs. stage III, and it correlated with worse OS in LUAD patients. These findings emphasize the potential role of these genes as prognostic biomarkers for NSCLC cases with BM.

In the last decade, researchers have increasingly focused on characterizing tumors using miRNA data, as these non-coding RNAs (ncRNAs) can significantly impact cancer development and progression. In general, a single miRNA may regulate multiple genes and thus multiple pathways, making it a powerful tool for biomarker use in disease diagnosis, risk assessment, and prognosis [29]. In NSCLC, several groups have reported different miRNAs associated with the disease and their correlations with clinicopathological factors. The study by Arora et al. identified miR-328 as promoting BM in NSCLC patients by increasing PKCα levels, which, in turn, increases cancer cell migration [30]. Similarly, miR-375, a brain-specific miRNA, has been downregulated in NSCLC patients with BM compared with those without BM [31]. Another report confirmed that miR-184 and miR-197 were upregulated in EGFR-mutated BM compared with primary tumors with EGFR mutation but without BM [32].

Interestingly, miR-23b has emerged as a promising candidate and novel biomarker in many cancers. In relation to NSCLC, miR-23b has been experimentally shown to function as a tumor suppressor, and its expression is downregulated in advanced tumor samples [33]. This is consistent with our study, which reported lower miR-23b expression in stage IV vs. stage II and further showed that this was associated with poor OS in LUAD patients. The other miRNA with a similar pattern was miR-429, whose expression levels were downregulated in stage IV vs. stage III in our results. miR-429 is not well studied, especially because few studies have elucidated its role in carcinogenesis. However, levels of miR-429 have been reported to decrease significantly in gastric carcinoma tissue specimens compared to adjacent non-tumor paired tissue samples [34], which is also consistent with our NSCLC data.

Conversely, we also reported that higher expression levels of miR-27a, miR-24-1, and miR-24-2 in stage IV vs. stage III LUSC patients correlated with poor OS. miR-27a plays an important role in tumorigenesis, proliferation, apoptosis, migration, and angiogenesis. In addition, increasing evidence has confirmed the clinical relevance of miR-27a in patient prognosis and has reported that it acts as an oncogene in many cancers [35,36]. Our results reveal an upregulation of miR-27a with tumor progression, suggesting that it can be employed as a marker for diagnosis and molecular targeted therapy in NSCLC patients. Similarly, the involvement of miR-24-1 and miR-24-2 in cancer progression has been reported in many studies [37,38,39]. Furthermore, reduced expression of miR-200b has been reported in LC patients [40]. Our study found decreased miR-200 b expression in stage IV vs. stage III and a further association with poor survival in LUSC patients. All these studies confirm the potential roles of various miRNAs in diagnosis, prognosis, and the identification of novel therapeutic avenues for NSCLC patients. However, their specific roles in inducing BM in NSCLC still need to be investigated experimentally.

While this study provides significant insights into the molecular drivers of LC metastasis to the brain, several limitations must be acknowledged. First, the use of stage IV TCGA samples as the primary discovery cohort introduces inherent biological heterogeneity. Stage IV disease encompasses a broad spectrum of distant metastatic sites, including the bone, liver, and contralateral lung. Consequently, the initial signatures identified may reflect mechanisms of general metastatic progression rather than those strictly specific to the brain microenvironment. To mitigate this, a hierarchical validation strategy was employed, prioritizing only those genes that were consistently dysregulated in both the TCGA discovery set and the brain-specific GSE161116 validation dataset. Second, the statistical power of certain analyses was constrained by the available sample sizes in public repositories. Specifically, the TCGA LUSC cohort contained only three stage IV samples (n=3). This small subgroup increases the uncertainty of the differential expression results for LUSC; therefore, these findings should be interpreted as exploratory and suggestive rather than definitive. Finally, the DEA were conducted using a nominal *p*-value threshold of <0.05 without formal adjustment for multiple comparisons, such as false discovery rate (FDR) correction. While FDR correction is standard for genome-wide studies to minimize type I errors, this study prioritized biological sensitivity in the discovery phase. The cross-cohort overlap between TCGA and GEO serves as a robust biological filter, as the probability of a false positive appearing consistently across independent patient populations and sequencing platforms is significantly reduced. Nevertheless, the lack of statistical correction remains a limitation, and the identified signatures should be viewed as high-priority candidates. Future research utilizing larger, site-specific metastatic datasets and functional experimental models will be essential to further distinguish brain-specific drivers from general metastatic markers and to validate these preliminary findings in a clinical context.

## 4. Materials and Methods

### 4.1. RNA-Seq and Microarray Data Extraction

#### 4.1.1. RNA-Seq Data Extraction

The inclusion and exclusion criteria for extracting RNA-seq data from the TCGA-LUAD and LUSC cohorts were previously discussed [41]. Only primary solid tumor samples across stages I–IV were retained in both cohorts. To ensure uniformity, the miRNA-seq data was also fetched with all its samples overlapping with the mRNA-seq samples for both cohorts.

#### 4.1.2. Microarray Data Extraction

We queried the NCBI-GEO [42] (https://www.ncbi.nlm.nih.gov/geo/, accessed on 20 March 2025) using “NSCLC brain metastasis” as a keyword for extracting NSCLC- and BM-associated mRNA expression profile. All the search results were filtered down by applying following inclusion criteria: (1) dataset must be of the type “expression profiling by array”, along with its samples belonging to “Homo sapiens”; (2) the dataset must include raw as well as pre-processed data; (3) the submission date of the dataset must be within the last five years (i.e., 2017 to 2022); (4) the dataset must contain greater than 25 samples; (5) the dataset must contain brain metastasis and NSCLC patient samples. We excluded any studies lacking case reports, review articles, abstracts, non-human samples, or cell-line-based experimental study designs.

### 4.2. DEA of Microarray and RNA-Seq Cohorts

#### 4.2.1. RNA-Seq DEA

We divided all the tumor sample expression data into three stage groups (i.e., stage IV vs. stage III, stage IV vs. stage II, stage IV vs. stage I) and back log-transformed all the miRNA as well as mRNA expression values to obtain raw integer counts because the originally downloaded values were already ${log}_{2}(x+1)$ transformed. The low-count Ensembl IDs in mRNA-Seq data were eliminated, followed by normalization and ${log}_{2}$ transformation through variance stabilizing transformation (VST) via the DESeq2 package, [43] whereas, for the miRNA-Seq data, we used the edgeR package [44,45] for obtaining normalized (upper quartile) and ${log}_{2}$-transformed expression values. Afterwards, we used the NOISeq package [46,47] with known batch settings to obtain batch-corrected mRNA/miRNA expression values, followed by z-score transformation using scale function. Probe-to-gene mapping for mRNA-seq data was performed as previously discussed [48]. Since the miRNA IDs were already present, there was no need to map miRNA-seq data. Next, we averaged the expression values for each gene/miRNA to handle duplicate entries and deleted low-variance (LV) genes (only 50%). We applied a two-sample unpaired *t*-test using the limma package [49] for obtaining *p*-values and log_2_ (fold change) values for all genes/miRNAs. The upregulated DEGs/DEMs were screened corresponding to a p-value<0.05 and ${log}_{2}\left(foldchange\right)>0,$ while the downregulated DEGs/DEMs were screened corresponding to a p-value<0.05 and ${log}_{2}\left(foldchange\right)<0$.

#### 4.2.2. Microarray DEA

The selected dataset’s series matrix file was extracted from GEO, followed by quality checks (QCs) for assessing data pre-processing. NOISeq package with known batch settings was used to obtain batch-corrected expression values, followed by z-score transformation using the scale function. Mapping probe ID to HUGO Gene Nomenclature Committee (HGNC) gene symbols was done using GEO2R. Next, we averaged gene expression values for duplicate handling and deleted 50% of LV genes. We applied a two-sample unpaired *t*-test using the limma package for obtaining *p*-values and ${log}_{2}(foldchange)$ values of all genes. The upregulated DEGs were screened corresponding to a p-value<0.05 and ${log}_{2}\left(foldchange\right)>0$, while the downregulated DEGs were screened corresponding to a p-value<0.05 and ${log}_{2}\left(foldchange\right)<0$.

### 4.3. Collection of DEMGs

The DEGs between stage IV vs. stage III, stage IV vs. stage II, and stage IV vs. stage I were identified for both the TCGA-LUAD and LUSC cohorts. Meanwhile, the DEGs between BM and NSCLC patient samples from GEO were identified. DEGs from each stage-wise category were individually overlapped with GEO DEGs and defined as DEMGs to identify robust metastasis-related genes involved in BM.

### 4.4. PPIN and OS Analyses

The union of DEMGs in both LUAD and LUSC cohorts was provided as input to the Search Tool for the Retrieval of Interacting Genes/Proteins (STRING) v11.5 database [50] (https://string-db.org/, accessed on 25 March 2025) to generate a PPIN with the highest confidence (i.e., interaction score >0.9), which was subsequently visualized in Cytoscape v3.10.0 [51] (https://cytoscape.org/, accessed on 30 March 2025). The DEMGs involved in PPIN and all DEMs were further subjected to OS analysis. The patients of LUAD and LUSC cohorts were dichotomized into high-, mid-, and low-expression groups based on z≥1.96, −1.96>z<1.96, and z≤−1.96 for DEMG/DEM expression separately. Log rank p-value<0.05 was considered as a statistically significant prognostic assessment threshold.

### 4.5. NSCLC Metastatic-Specific Three-Node miRNA FFL Analysis

To compile regulatory interactions among TFs, miRNAs, and mRNAs, a three-node miRNA FFL specific to metastatic NSCLC was constructed as follows:

#### 4.5.1. TF-mRNA Regulation

We accessed the ChEA v3.0 database [52] (https://maayanlab.cloud/chea3/, accessed on 1 April 2025) and the TRRUST 2019 library within the Enrichr database [53,54] (https://maayanlab.cloud/Enrichr/, accessed on 2 April 2025) in order to fetch significant (p-value<0.01) human TFs regulating our prognostic DEMGs for compiling the TF-mRNA regulation pairs. All the TFs were further validated in the literature, and only those linked to NSCLC were retained.

#### 4.5.2. miRNA-mRNA Repression

We accessed the miRWalk v3.0 database [55] (http://mirwalk.umm.uni-heidelberg.de/, accessed on 5 April 2025) in order to compile the miRNA-mRNA repression pairs. Significant miRNAs (having score>0.95, binding only on 3′UTR region, and having bindinggap=1) repressing our prognostic DEMGs were collected from miRWalk.

#### 4.5.3. miRNA-TF Repression

The NSCLC-validated TFs obtained from TF-mRNA regulation pairs were further utilized for fetching the miRNA-TF pairs. TFs repressed by our prognostic DEMs were extracted from miRWalk with the previously described thresholds. Lastly, all three molecular interaction pairs (i.e., TF-mRNA, miRNA-mRNA, miRNA-TF) were altered with respect to validated TFs and prognostic DEMGs/DEMs. All these pairs were merged to establish an NSCLC metastatic-specific three-node miRNA FFL and visualized utilizing Cytoscape v3.10.0.

### 4.6. Mutational Analysis of FFL Elements

We accessed the cBioPortal (https://www.cbioportal.org/, accessed on 16 April 2025) database [56] to explore the genomic alterations in our TFs and DEMGs within our FFL. We selected the LUAD (TCGA, Firehose Legacy) cohort in cBioPortal and included only stage-specific samples that contained our FFL elements (i.e., TFs and DEMGs).

## 5. Conclusions

In conclusion, our study provides a comprehensive overview of the complex molecular landscape of NSCLC brain metastasis. While existing research has often focused on isolated genomic drivers, our work successfully decodes the higher-order regulatory architecture between *CD44*, miR-23b-3p, and TFs. This three-node FFL analysis uncovers potential genomic drivers that may play crucial roles in NSCLC BM, effectively bridging the gap between bioinformatics discovery and clinical stratification. Furthermore, integrating mutational data with survival outcomes across LUAD and LUSC cohorts provides a robust, multidimensional validation of these biomarkers. By establishing the prognostic significance of this regulatory circuit, our study offers valuable insights for the discovery of novel therapeutic targets in the management of NSCLC metastasis. However, while this integrative approach offers a high-confidence genomic roadmap, further in vitro and in vivo experimentation remains imperative to justify and establish the role of these specific NSCLC biomarkers in BM pathogenesis.

## Figures and Tables

**Table 1:** Clinical patients’ information from the TCGA-LUAD cohort.

Characteristics	Stage I (*n* = 217)	Stage II (*n* = 89)	Stage III (*n* = 62)	Stage IV (*n* = 18)
Age				
<65	94	44	26	14
≥65	123	45	36	04
Race				
Asian	04	01	-	02
White	190	74	55	14
Black	23	14	07	02
Gender				
Male	82	47	25	12
Female	135	42	37	06

**Table 2:** Clinical patients’ information from the TCGA-LUSC cohort.

Characteristics	Stage I (*n* = 173)	Stage II (*n* = 121)	Stage III (*n* = 50)	Stage IV (*n* = 03)
Age				
<65	51	48	22	-
≥65	122	73	28	03
Race				
Asian	02	04	02	01
White	157	111	43	02
Black	14	06	05	-
Gender				
Male	120	88	36	03
Female	53	33	14	-

**Table 3:** Details of DEGs across the TCGA-LUAD and the LUSC cohorts.

Stage-Wise Groups	Total DEGs	Upregulated	Downregulated
LUAD			
stage IV vs. stage III	842	326	516
stage IV vs. stage II	647	431	216
stage IV vs. stage I	959	415	544
LUSC			
stage IV vs. stage III	455	267	188
stage IV vs. stage II	353	205	148
stage IV vs. stage I	250	159	91

**Table 4:** Details of DEMs across the TCGA-LUAD and the LUSC cohorts.

Stage-Wise Groups	Total DEMs	Upregulated	Downregulated
LUAD			
stage IV vs. stage III	05	02	03
stage IV vs. stage II	17	05	12
stage IV vs. stage I	10	08	02
LUSC			
stage IV vs. stage III	17	10	07
stage IV vs. stage II	09	02	07
stage IV vs. stage I	11	01	10

**Table 5:** Details of age and gender of patients contributing to stage-wise DEG analysis.

Stage-Wise Groups	Mean Age	Minimum Age	Maximum Age	Gender Ratio (Male/Female)
LUAD				
stage IV vs. stage III	63.67	40	83	37/43
stage IV vs. stage II	63.42	40	84	59/48
stage IV vs. stage I	65.10	38	88	94/141
LUSC				
stage IV vs. stage III	65.16	44	84	39/14
stage IV vs. stage II	66.79	41	84	91/33
stage IV vs. stage I	68.34	40	85	123/53

## Data Availability

The raw HTSeq count datasets of TCGA-LUAD and LUSC used in our study were downloaded from UCSC Xena Browser available at https://xenabrowser.net/datapages/?dataset=TCGA-LUAD.htseq_counts.tsv&host=https%3A%2F%2Fgdc.xenahubs.net&removeHub=https%3A%2F%2Fxena.treehouse.gi.ucsc.edu%3A443, accessed on 20 March 2025 and https://xenabrowser.net/datapages/?dataset=TCGA-LUSC.htseq_counts.tsv&host=https%3A%2F%2Fgdc.xenahubs.net&removeHub=https%3A%2F%2Fxena.treehouse.gi.ucsc.edu%3A443, accessed on 20 March 2025. Microarray dataset used in our study was downloaded from NCBI-GEO available at https://www.ncbi.nlm.nih.gov/geo/query/acc.cgi?acc=GSE161116, accessed on 20 March 2025.
